# Contamination Sources and Transmission Routes for *Campylobacter* on (Mixed) Broiler Farms in Belgium, and Comparison of the Gut Microbiota of Flocks Colonized and Uncolonized with *Campylobacter*

**DOI:** 10.3390/pathogens10010066

**Published:** 2021-01-13

**Authors:** Karolien Hertogs, Annelies Haegeman, Dries Schaumont, Philippe Gelaude, Lieven De Zutter, Jeroen Dewulf, Marc Heyndrickx, Geertrui Rasschaert

**Affiliations:** 1 Flanders Research Institute for Agriculture, Fisheries and Food (ILVO), 9820 Merelbeke, Belgium; karolien.hertogs@ugent.be (K.H.); annelies.haegeman@ilvo.vlaanderen.be (A.H.); dries.schaumont@ilvo.vlaanderen.be (D.S.); marc.heyndrickx@ilvo.vlaanderen.be (M.H.); 2 Department of Reproduction, Obstetrics and Herd health, Faculty of Veterinary Medicine, Ghent University, 9820 Merelbeke, Belgium; jeroen.dewulf@ugent.be; 3 Animal Health Care Flanders (DGZ), 8820 Torhout, Belgium; gelaudephilippe@gmail.com; 4 Department of Veterinary Public Health and Food Safety, Faculty of Veterinary Medicine, Ghent University, 9820 Merelbeke, Belgium; lieven.dezutter@ugent.be; 5 Department of Pathology, Bacteriology and Poultry Diseases, Faculty of Veterinary Medicine, Ghent University, 9820 Merelbeke, Belgium

**Keywords:** *Campylobacter*, broilers, farm, partial thinning, metabarcoding

## Abstract

Biosecurity seems to be the most promising tool for *Campylobacter* control on poultry farms. A longitudinal molecular epidemiological study was performed during two production cycles, in which the broilers, the poultry house, and the environment of 10 (mixed) broiler farms were monitored weekly. Cecal droppings from the second production cycle were also used for 16S metabarcoding to study the differences in the microbiota of colonized and uncolonized flocks. Results showed that 3 out of 10 farms were positive for *Campylobacter* in the first production cycle, and 4 out of 10 were positive in the second. Broilers became colonized at the earliest when they were four weeks old. The majority of the flocks (57%) became colonized after partial depopulation. Before colonization of the flocks, *Campylobacter* was rarely detected in the environment, but it was frequently isolated from cattle and swine. Although these animals appeared to be consistent carriers of *Campylobacter*, molecular typing revealed that they were not the source of flock colonization. In accordance with previous reports, this study suggests that partial depopulation appears to be an important risk factor for *Campylobacter* introduction into the broiler house. Metabarcoding indicated that two *Campylobacter*-free flocks carried high relative abundances of *Megamonas* in their ceca, suggesting potential competition with *Campylobacter*.

## 1. Introduction

Both globally and nationally, campylobacteriosis is a recognized health risk. In the industrialized world, *Campylobacter* species appear to be the main cause of human gastroenteritis. Within Europe, 246,541 cases are diagnosed each year, making *Campylobacter* the most commonly reported bacterial food pathogen [1]. It was estimated that 50 to 80% of the human *Campylobacter* cases originate from the poultry reservoir [2], mainly via the handling or consumption of contaminated poultry meat [3]. Based on these data, the broiler industry appears to be responsible for a large proportion of the human *Campylobacter* infections. Intervention at the level of primary production may potentially reduce the number of human infections. Calculations of Messens and co-authors [4] indicated that a 10-fold reduction in *Campylobacter* counts in the chicken ceca may lead to a 55% reduction in the human campylobacteriosis cases.

Identified risk factors for *Campylobacter* introduction in the poultry house are the presence of other animals on the farm such as rodents, insects, wild birds, pets, and other animals. Other risk factors are the slaughter age, flock size, age and number of poultry houses, farm personnel, insufficient biosecurity including partial depopulation, farm equipment, and transport vehicles [5]. Even though poultry is the major reservoir for *Campylobacter*, other farm animals such as cattle and swine are also seen to be carriers of *Campylobacter* [6,7]. Therefore, mixed farms are assumed to be more at risk for *Campylobacter* spread to the broiler house [8]. In Belgium, 20% of the poultry farms breed cattle in addition to poultry, and 10% raise pigs in addition to poultry (DGZ, personal communication 2020).

Interventions such as vaccination and immunization, phage therapy, and feeding strategies such as adding prebiotics to the feed or additives to the drinking water have only shown limited success in *Campylobacter* reduction [9]. Currently, the most promising intervention strategy against *Campylobacter* seems to be strict biosecurity management [10,11]. In Denmark, for example, the use of fly screens led to a reduction in the *Campylobacter* prevalence by 30% [12,13]. Although risk factors for *Campylobacter* introduction are presumably similar between various countries, climate conditions can have a major impact on the survival and occurrence of *Campylobacter* in the environment, because it is a thermotolerant bacterium. Therefore, regional differences in the presence and spread of this pathogen may be present.

Besides differences in biosecurity approaches and hygiene practices, biological variation between flocks may be an important factor. An important cause of biological variation is the composition of the gut microbiota. Competitive exclusion of bacteria present in the gut can influence *Campylobacter* development in the broiler’s gastrointestinal tract [14], and consequently may act as a pre-disposing factor for flock colonization.

The aim of this study was to reveal the possible contamination sources and transmission routes on both mixed and exclusive broiler farms, as well as identifying cecal microbial composition of both *Campylobacter*-colonized and *Campylobacter*-free flocks. This knowledge may be useful for the implementation of biosecurity measures and may provide insights into individual flock susceptibility.

## 2. Results

No *Campylobacter* could be isolated from the broiler house immediately after cleaning and disinfection. At that time, *Campylobacter* was only isolated outside the broiler house in the wider environment, such as in puddles, carcass containers, and in the stables for the other livestock animals (if present). During rearing of the broilers, *Campylobacter* was only detected in the broiler house or in the anteroom if the broilers were colonized themselves. This means that contamination of the direct environment in the broiler house and colonization of the broiler flock was detected simultaneously.

In total (production cycle 1 + 2), 7 out of 20 flocks became colonized with *Campylobacter*, and farm A was affected twice (with two different *C. jejuni* strains). During the first cycle, three flocks were found to be positive (farms A, B and C). During the second cycle, four flocks were *Campylobacter*-colonized (farms A, D, G and H). Additionally, 4 of the 10 farms managed to keep the flocks *Campylobacter*-free during both production cycles (E, F, I, J).

**Figure 1:**
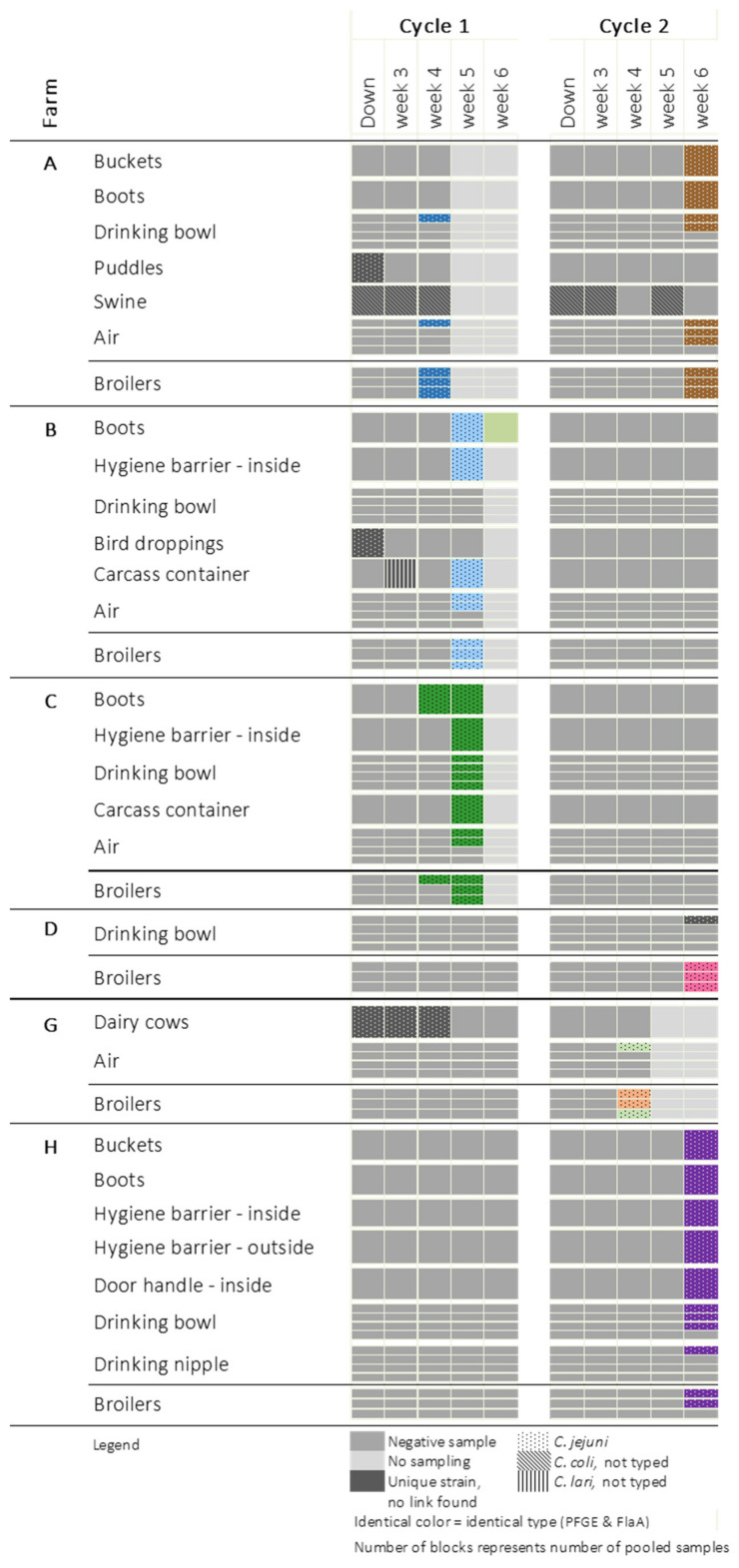
Occurrence of *Campylobacter* at farms where at least one broiler flock became colonized.

**Figure 2:**
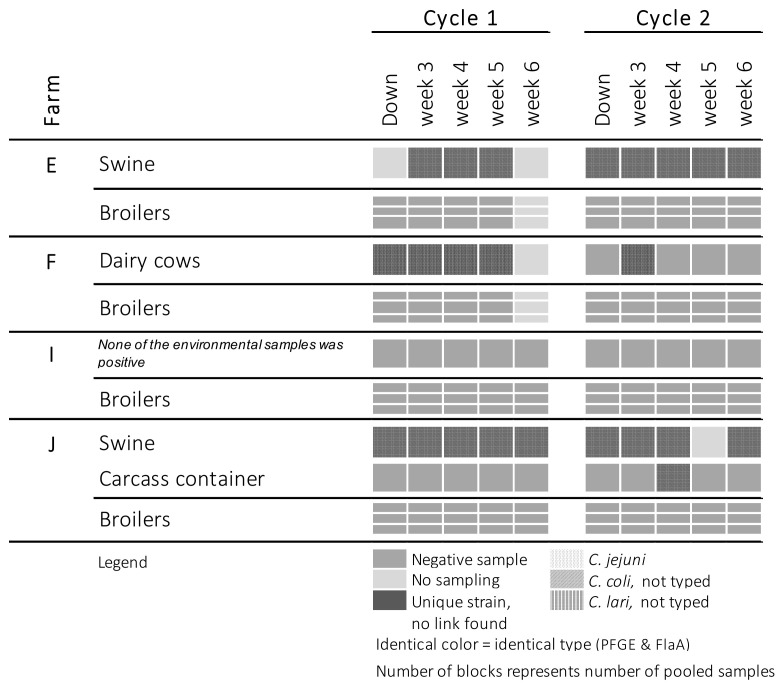
Occurrence of *Campylobacter* at farms where both broiler flocks remained *Campylobacter*-negative.

Figure 1 and Figure 2 show the places where *Campylobacter* was detected on farms: where the broilers were *Campylobacter*-positive for at least one cycle (Figure 1) and where the broilers remained free of *Campylobacter* (Figure 2). An overview of all sampled places can be found in the Supplementary Table S1. Flocks identified as *Campylobacter*-colonized were all colonized with *C. jejuni*. The colonization level varied between 5 and 6 log cfu/g cecal droppings. Colonization was not detected before broilers reached an age of four weeks. Of the seven colonized flocks, four became positive at five to six weeks of age, after partial depopulation. Once the broilers were colonized, *Campylobacter* was widely present in the broiler house and anteroom. In five flocks originating from four farms, *Campylobacter* was isolated from the air present in the broiler house. For four flocks, *Campylobacter* was detected in the water of the drinking cups/bowls, and at one sampling event it could also be isolated from the drinking nipples. Campylobacters found in the poultry house (e.g., drinking bowls, air) or in the anteroom (e.g., floor, boots) were in most cases identical to the strain isolated from the colonized birds. *Campylobacter* was isolated twice from the buckets. In four cases, *Campylobacter* strains identical to those colonizing the broilers were isolated from boots, and in three cases they were also detected on the internal side of the hygiene barrier (inside HB), where those boots are stored and exchanged. The external side of the hygiene barrier (outside HB) tested positive in one anteroom belonging to farm H. At that farm, the outdoor door handle of the anteroom was also found to be contaminated with *Campylobacter*.

In contrast to the inside of the broiler house and anteroom, contamination of the outside environment often occurred independently from the *Campylobacter* status of the flock. *C. jejuni* was once isolated from a puddle on farm A, but this was before the birds became colonized, and was also a different strain than the one found in the colonized broilers. *C. jejuni* was also isolated twice from puddles on farm J, where the broilers did not become colonized. *C. jejuni* was isolated from wild bird droppings on farm B. Again, this was a different strain from the strain isolated from the colonized flock. *Campylobacter* was detected three times on the carcass containers (farms B, C and J). *C. lari* was isolated from the carcass container present at farm B.

Cattle (farms F and G) and swine (farms A, E and J) were colonized with *C. jejuni* and *C. coli*, respectively. However, strains could not be matched with the strains isolated from the broilers. *Campylobacter* was not constantly detected in dairy herds. Dairy cows present on farm G were consistently shedding *C. jejuni* during the first cycle. However, during the second cycle, *Campylobacter* could not be demonstrated in samples originating from the same herd. On this farm, broilers were only found to be *Campylobacter*-positive during this second cycle. Moreover, the *Campylobacter* strain isolated from the broilers did not match the strains found in the dairy herd during the first production cycle. On farm F, the dairy cows were shedding campylobacters during both production rounds, but the broilers remained *Campylobacter*-free. On the three broiler–pig farms, pigs were colonized with *C. coli* during both production cycles, whereas the broilers were colonized with *C. jejuni* (farm A) or remained *Campylobacter*-free (farms E and J). 

When comparing results of 16S metabarcoding (only production cycle 2) and cultivation, cultivation appeared to be a more accurate technique to detect *Campylobacter* in cecal samples. Some (culture confirmed) *Campylobacter*-positive samples remained undetected using 16S metabarcoding, regardless of the *Campylobacter* counts. In the second production cycle, four flocks were found to be positive by classic culture: flocks from farms A, D and H at the age of six weeks, and a flock from farm G at four weeks. Each time, all pooled samples were positive by classical culture. With metabarcoding, the same flocks were identified as being *Campylobacter*-colonized, but only one to four out of the five samples appeared positive.

**Figure 3:**
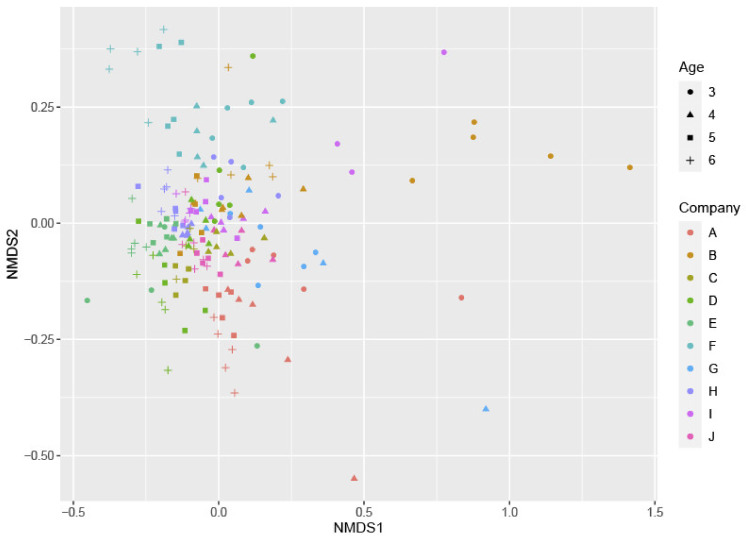
Non-metric multidimensional scaling (NMDS) profile of pairwise community dissimilarity (Bray-Curtis) indices of 16S sequencing data of all samples. The shapes represent different ages of the flocks, and colors indicate different farms.

However, the results of the metabarcoding analysis were relevant regarding the richness and composition of the microbiome. In each flock, bacterial richness (the number of various taxonomic groups present) was dependent on age, because the diversity of the gut microbiome of three-week-old broilers was significantly smaller (*p* < 0.001) than the microbiome of older broilers (Supplementary Figure S1). When comparing flocks from different farms, there were no significant differences found in bacterial richness, although the flock at farm F tended to show a higher number of taxa compared to the other farms. There was no indication that the richness was different in the case of a *Campylobacter* colonization (*p* = 0.894) based on the culture status (*Campylobacter*-positive versus *Campylobacter*-negative samples). Nevertheless, even though there were no differences in bacterial richness between colonized and non-colonized flocks, results showed that both the farm of origin (*p* = 0.001) and the age of the broilers (*p* = 0.001) did have a significant influence on the composition of the microbial community and the abundance of some taxonomic groups in the broilers’ ceca (Figure 3). Overall, the families of *Lactobacillaecae*, *Ruminococcaeae*, and *Lachnospiraceae* were most abundant in the cecum. When comparing the microbial community of broilers, some genera significantly differed between age group 3 and 4 (Table 1). The genera of *Staphylococcus*, *Corynebacterium*, *Brachybacterium*, *Weisella*, *Bacteroides* and *Lactobacillus* were significantly more present at younger ages (in descending order), as indicated by the negative log2-fold changes (*p* < 0.05) at an age of four weeks compared to an age of three weeks. In contrast, the positive log2-fold change values showed that the genera of *Streptococcus, Fusicatenibacter*, and *Subdoligranulum* had a significantly increased relative abundance in four-week-old broilers compared to three-week-old broilers. However, values for the latter two groups were very low, indicating only a small difference.

Similar statistical analyses also showed some significant differences between microbial genera of non-colonized versus colonized flocks (Table 2). We considered genera to be significant at an abundance of >0.5% only. One of these was the *Streptococcus* bacterium, which was seen to occur more often when flocks were colonized. In contrast, *Subdoligranulum*, *Fusicatenibacter* and *Megamonas* showed a positive log2-fold change, meaning that they occurred more often in *Campylobacter*-free flocks. *Megamonas spp*. were found in two *Campylobacter*-negative flocks (present in farms E and F). These bacteria occurred within this group in an abundance >8% of the total microflora and appeared to be absent in *Campylobacter*-positive flocks. Remarkably, the presence of this genus was seen to increase with age, and rose strongly from five weeks of age onwards. Furthermore, *Sutterella*, *Helicobacter*, and *Barnesiella* were also significantly more present in *Campylobacter*-negative flocks, but the mean percentage of occurrence was relatively low compared to *Megamonas* spp.

## 3. Discussion

In this Belgian study, 35% of the flocks became colonized with *Campylobacter*. According to data from the European Food Safety Authority (EFSA) [1], 26% of the EU broiler flocks raised in 2018 were colonized with *Campylobacter*. This is in accordance with our findings. In earlier Belgian studies, the prevalence of *Campylobacter* in flocks was between 65 and 80% at slaughter age [15,16,17]. A possible explanation for the lower incidence in the present study may be the period of extremely dry weather that coincided with the time of sampling (May to August 2018); *Campylobacter* is known to be susceptible to dry conditions [18]. In addition, Belgium had to deal with an outbreak of Newcastle disease during this period. This increased the awareness of biosecurity implications among poultry farmers, probably resulting in an improved application of biosecurity measures and hygiene practices, possibly with a lower *Campylobacter* prevalence as a consequence.

Although the sampling was specifically designed to identify the initial contamination sources for flock colonization, we were unable to unambiguously identify them in the present study. Contamination of the environment and colonization of the broilers were detected at the same sampling point; therefore, it was impossible to distinguish the direction of contamination. However, the observations indicate the direction of a rapid spread of *Campylobacter* throughout the poultry house, as reported before [19,20]. Both studies demonstrated that one individual colonized bird may lead to the colonization of nearly all birds within one week. Molecular typing of isolates in this current study also showed that, once present, *Campylobacter* is spread via the air, litter, and water, and leads to the contamination of boots, buckets, the anteroom, and the exterior of the poultry house, such as the carcass container. These findings are in agreement with the results of previous studies [17,21,22,23,24,25]. According to Søndergaard et al. [26], air samples could test positive before broiler colonization is detected, making air samples a good predictor for *Campylobacter* colonization. This hypothesis could not be confirmed in the present study.

In all cases, *Campylobacter* contamination of the floor ended at the hygiene barrier, with the exception of farm H where the barrier consisted of a single line drawn on the floor instead of a physical barrier. At this farm, the “clean” side of the hygiene barrier was also contaminated with *Campylobacter*. This indicates the advantages of this small biosecurity measure when properly applied. Moreover, it is seen that buckets, boots and the carcass container may be contaminated with *Campylobacter* and can potentially operate as a transmission vehicle in and outside the broiler house.

One of the aims of this study was to investigate whether mixed farms would be at higher risk for *Campylobacter* introduction in broiler houses compared to farms that only held poultry at their site. In our study, farm animals were not a risk factor for *Campylobacter* introduction. Mixed and single poultry farms seemed to show an equal level of *Campylobacter* prevalence, although this is based on a low number of flocks. This could either be due to applying strict biosecurity, or to a lower susceptibility of the broilers to *Campylobacter* strains that colonize pigs or cows. Broilers seemed to become colonized with *C. jejuni* more frequently compared to *C. coli* in this current study; no *C. coli* was found in the flocks. This is in accordance with findings of other studies, as reviewed by Hermans et al. [5]. According to Carrique-Mas et al. [27] there does not seem to be an additional risk for *C. coli* colonization in poultry flocks that are exposed to pigs on their site. On the other hand, transmission of *C. jejuni* genotypes from herds of cattle to broiler meat was already demonstrated by Ragimbeau and co-workers [28]. Although Patriarchi et al. [29] consider the presence of cattle as an underestimated risk for flock colonization based on molecular typing, a direct link between the cattle herd and broiler flock present on one farm was not identified in that study. Cows seem to be intermittent *Campylobacter* shedders, demonstrated both in the present study and in previous studies [30]. Contrary to the literature, the results of our study do not indicate cattle or swine to be a transmission source for broiler flocks.

Even though we were unable to indicate the source of contamination, we hypothesize that partial depopulation may have been the source of *Campylobacter* introduction in half of the cases—this was shown in a previous study by our group when monitoring the same farms [31]. The majority of colonizations (57%) occurred at five to six weeks of age, after a proportion of the broilers had been transported from the poultry farm to the slaughterhouse. During this so-called thinning procedure, containers (originating from the slaughterhouse) are brought into the poultry house and a catching crew is responsible for loading the chickens. It has already been demonstrated that the catching crew and materials used during this procedure (such as containers and transportation vehicles) can be contaminated with *Campylobacter* [32], and form a risk for pathogen transfer. In the study of Ridley et al., strains detected at the time of thinning were also found in the ceca of the broilers that remained in the poultry house for another week [33]. 

The lack of evidence for the source of earlier colonizations could potentially be explained by a viable-but-non-culturable state (VBNC) of the *Campylobacter* species, as described previously [34]. When *Campylobacter* thrives under non-optimal environmental conditions, it can be present in a state in which it is not detectable by culturing but is still able to cause colonization. One hypothesis may be that *Campylobacter* in environmental samples, taken in this current study, was present in a VBNC state, and therefore was not detected via cultivation. Once the broiler flock became colonized, there was a constant flow of *Campylobacter* to the environment, making the bacteria more detectable in environmental swab samples after enrichment.

This study also aimed to determine the differences in microbiota of *Campylobacter*-colonized and *Campylobacter*-negative flocks. Although we did find a significant difference between both groups, these results should be interpreted with care. Firstly, antimicrobials (mainly amoxicillin, doxycycline, and lincomycin in combination with spectinomycin) were used in all flocks during the second production round, with the exception of farm C. Clearly, this influenced the composition of the microbiota. Secondly, the farm effect was significant, suggesting that each farm flock had its own microbiome composition, making it more difficult to draw overall conclusions. Additionally, we found that the microbiome richness and composition of three-week-old broilers was significantly different from older age groups. The increase in bacterial richness with broiler age has been described by Gong et al. [35]. Nevertheless, by including age and farm as co-variables in our statistical models, some genera did show significant differences between *Campylobacter*-positive and *Campylobacter*-negative flocks. The genus *Bacteroides*, for example, was shown to be more present in non-colonized flocks. However, this was mainly a consequence of only one flock that carried high numbers of *Bacteroides plebeius* in their ceca. More interesting is the increased abundance of *Megamonas* in two *Campylobacter*-negative flocks, which was undetected in all colonized flocks. In addition, this genus was seen to increase with age, and was highly abundant at an age of five weeks, which corresponded to the most common period that *Campylobacter* colonized the ceca. *Megamonas hypermegas* (formerly known as *Bacteroides hypermegas*) was previously described as being competitive with *Salmonella* in vitro [36], which makes this bacterium a potential probiotic candidate against (food) pathogens. The effect would be due to the breakdown of non-starch polysaccharides into short-chain fatty acids (SCFAs), causing a lower pH [36,37,38]. This can therefore create a less optimal environment for food pathogens, such as *Salmonella* (with potentially identical effects for *Campylobacter*) that could suppress the growth of these bacteria within the broiler intestinal tract. Out of all SCFAs, *Megamonas* is seen to mainly produce propionic acid [39]. Additionally, results from an experiment conducted by Gonzalez-Fandos et al. demonstrated propionic acid to be effective in inhibiting *C. jejuni* on contaminated broiler skin samples [40], and a study by Scupham et al. showed *Megamonas hypermegale* strains to suppress *C. jejuni* strains in turkeys [41]. More in-depth research is needed in order to draw conclusions on opportunities of the usage of *Megamonas* representatives as a probiotic against *Campylobacter* in broilers under field conditions.

In conclusion, results of this current study indicate that half of the *Campylobacter* colonizations occurred in the last week of the rearing period, and initial contamination sources could not be identified. Thinning may be responsible for *Campylobacter* introduction in the broiler house in many cases, because this practice was performed before *Campylobacter* appeared. Outcomes of this current study suggest the need for further research in this area. Furthermore, mixed farms were not considered as a risk factor for *Campylobacter* colonization of the broilers. Finally, *Megamonas* could act as a probiotic strain to reduce *Campylobacter* colonization.

## 4. Materials and Methods

### 4.1. Sampling

The choice of places to sample on the farms was based on results of studies conducted in the European Union regarding the potential risk factors for *Campylobacter* introduction in broiler houses [13,17,33,42,43,44,45,46,47].

This current study was performed from April 2018 to January 2019 on 10 Belgian broiler farms (A–J), selected either based on their prior *Campylobacter* status and/or voluntary registration. The first production cycle was sampled from April to August 2018. During this period, the mean temperature was considered as exceptional. The mean monthly temperatures increased from 13 °C (April) to 22.1 °C (July) which was a few degrees higher than normal. This period was also dryer than normal. The second production cycle took place from the end of July 2018 until January 2019. The mean monthly temperatures decreased from 22.1 °C (July) to 3 °C (January), and the monthly mean temperatures were considered as normal during this period [48]. Flock size in the poultry house ranged from 18,000 to 100,000 birds. Five farms were mixed, with either cattle (farm F and G) or swine (A, E and J) raised in combination with broilers. One broiler house per site was selected for sampling, where the broilers and their environment were monitored weekly during 2 non-consecutive production cycles. One production cycle consisted of approximately 42 days, and thinning was performed around 35 days of age. 

The first sampling cycle took place during the down period after cleaning and disinfection of the broiler house. At that time, sampling included the anteroom and the broiler house with all fomites, flies, and beetles if present, wet spots on the floor, cracks in the floor and walls, the drinking system, and the air. Outside the broiler house, puddles, wild bird droppings, and the carcass container were all sampled. In addition, manure from cattle or swine was collected (Table 3). The floor of the anteroom was sampled by swabbing a surface (equal to an A4-size sheet of paper) on both sides of the hygiene barrier with 3M sponge sticks (Led Techno, Heusden-Zolder, Belgium), pre-moistened with 10 mL Bolton Broth (CM0983B, Oxoid Basingstoke, UK) without selective supplements. Other fomites present in the anteroom (boots, buckets, and the sink) were sampled in exactly the same way. Door handles were sampled using sterile cotton swabs pre-moistened with Bolton Broth. Flies were caught in the anteroom on sticky tape applied for approximately one week, then transferred to a sterile bag, while beetles were caught in the poultry house with sterile tweezers and stored in a sterile container for transportation to the lab. Beetles were frozen at −80 °C for approximately 15 min before being crushed. Cracks in the floor and walls, as well as the drinking system, were sampled with pre-moistened sterile cotton swabs. Cracks were sampled using one cotton swab for each side of the wall and one for the floor, making one pool. For drinking cups and nipples, 1 cotton swab was used to sample 5 units. This was repeated in order to collect pools of samples of both nipples and cups, originating from every area of the poultry house. In addition, 4 air samples were taken in the poultry house by the use of a Reuter Centrifugal Air Sampler (Hycon, Biotest AG, Dreieich, Germany), for which a total volume of 400 L was tested, and air strips were filled with rapid *Campylobacter* agar medium (RCA) (3564295, BioRad, California, USA). Dry sponge sticks were used for sampling of puddles, both inside and outside of the broiler house. Cattle and swine were screened for the presence of *Campylobacter* by using one pair of overshoes per animal species. The (empty) carcass container was also sampled for *Campylobacter* using a pre-moistened sponge stick.

Subsequently, weekly consecutive samplings were performed, starting from 3 weeks of age until broilers became colonized, or until slaughter age. Both excreta and environmental samples were screened for the presence of *Campylobacter* (for sampling design see Table 4). Three pools of 10 cecal droppings were collected per broiler house per time point during the first cycle. For the second cycle, 5 pools were collected. In addition to the broiler house (where broilers, the air, and drinking systems were sampled) the external environment (carcass container, puddles, manure from other farm animals and bird droppings if present) and the anteroom (boots, buckets, floor at both sides of the hygiene barrier, the sink, door handles and flies if present) were also sampled using the same protocol as described above. All samples were transported to the laboratory in a closed, refrigerated box. For the second cycle, aliquots of all 5 pools of cecal droppings were flash frozen in liquid nitrogen and stored at −80 °C before applying metabarcoding.

### 4.2. Microbiological Analysis

For culturing, 3 pools of cecal droppings were used. They were used both for enumeration, as shown in the Supplementary Table S1, and detection after enrichment. For enumeration, dilutions of 10^−1^, 10^−3^ and 10^−5^ were prepared in 0.1% peptone water (Biotrading, Keerbergen, Belgium), and 0.1 mL of each dilution was plated on rapid *Campylobacter* agar (RCA; 3564295, BioRad, Hercules, CA, USA) plates with rapid *Campylobacter* supplement (3564296, BioRad, Hercules, CA, USA), while for detection 90 mL Bolton Broth supplemented with Modified Bolton Broth Selective Supplement (Oxoid) and 5% of defibrinated horse blood (Intermed, Brussels, Belgium) was added to 10 g cecal droppings. Overshoes worn in the barn of the other farm animals present were directly plated on RCA, as well as enriched in 225 mL Bolton Broth. Environmental samples were only enriched. In total, 90 mL of Bolton Broth was added to the sponge sticks, 10 mL to the cotton swabs, and 225 mL to the overshoes. All sponge samples and overshoes were homogenized using a stomacher (Interscience, St. Nom la Bretèche, France).

One loopful of each enrichment broth was transferred after 24 h and 48 h of incubation onto RCA. After incubation of the RCA media for both 24 h and 48 h, plates were examined for the presence of suspected *Campylobacter* colonies. In the case of enumeration, the number of suspect colonies were counted. The mean value of all countable plates (<300 cfu/plate) was calculated, and expressed in number of cfu/g. Three suspect colonies were purified by streaking onto modified charcoal cefoperazone deoxycholate agar (mCCDA) (CM0739B, Oxoid). All incubations occurred under microaerophilic conditions (10% CO_2_, 5% O_2_, 85% N_2_) at a temperature of 41.5 °C. Presumptive *Campylobacter* isolates were suspended in 1 mL lysed horse blood and stored at −80 °C for later molecular analyses. A maximum of either 2 (enrichment at 24 h and 48 h) or 3 (direct plating and enrichment) isolates per sample were stored.

### 4.3. Molecular Analysis

Presumptive *Campylobacter* colonies were cultured on mCCDA plates, and lysates were made by resuspending a few of the colonies in 100 µL of sterile water and heating it at a temperature of 95 °C for 10 min. These lysates were screened with *Campylobacter*-specific PCRs [49,50]. All *C. jejuni* isolates were further typed by means of Flagellin gene A PCR/restriction fragment length polymorphism (FlaA analysis) and pulsed field gel electrophoresis (PFGE) according to the harmonized protocol from PulseNet [51,52]. SmaI was used as the main restriction enzyme. An additional PFGE analysis was performed with KpnI on isolates for which either no fingerprint was obtained by means of FlaA analysis or PFGE (SmaI), or for isolates originating from different farms that showed identical patterns with SmaI. Similarity between fingerprints was analyzed using Bionumerics Software (Applied Maths, Sint-Martens-Latem, Belgium). Fingerprints were matched based on the Dice coefficient, with a band-matching tolerance of 1% and an optimization coefficient of 1%. Cluster analysis was performed by the use of an unweighted-pair group method with mathematical averages (UPGMA). Identical fingerprints, based on visual examination, were assigned an identical color (Figure 1), which represented an identical pattern between *Campylobacter* isolates for both FlaA analysis and PFGE (SmaI and/or KpnI if tested).

### 4.4. 16 S Metabarcoding

#### 4.4.1. DNA Extraction, Library Preparation and Sequencing

All five pools of cecal droppings originating from the second production cycle were used for DNA extraction (*n* = 178) using the PowerSoil Pro extraction kit (Qiagen, Hilden, Germany). DNA yield was measured by use of nanodrop spectrophotometry (ThermoFisher Scientific, Waltham, MA, USA) and Quantus fluorometry (Promega Corporation, Fitchburg, WI, USA). Samples were diluted according to their total DNA yield, as measured by the Quantus double-stranded DNA assay. If >100 ng/µL: 1:5 dilution, if <100 ng/µL: 1:2 dilution, if <10 ng/µL: no dilution. Subsequently, amplicon sequencing of the bacterial V3–V4 region of the 16S rRNA gene was performed as described by the Illumina protocol and the primers of Klindworth et al. [53] on an Illumina MiSeq sequencer with 2 × 300 bp reads (Admera Health, South Plainfield, NJ, USA). The sequencing data have been deposited with links to the BioProject accession number PRJNA643676 in the NCBI SRA database.

#### 4.4.2. Processing of Sequence Reads and Downstream Analysis 

The entire processing pipeline, from reads to count table to statistical analyses, was performed in R v3.6.1 [54], run in RStudio 1.1.447 [55]. Reads were read into R and primers were removed using the ShortRead package [56]. Next, the reads were quality filtered and trimmed using the “filterAndTrim” function from the dada2 package [57]. Forward and reverse reads were trimmed to 280 bp and 210 bp, respectively, and quality filtering was performed with a maximum expected error of 2 for the forward and 4 for the reverse reads. The error correction model from dada2 was used to correct the resulting reads, after which the reads were merged and count tables from the amplicon sequence variants (ASVs) were calculated. Taxonomy was assigned using the IdTaxa function of the DECIPHER package [58], using SILVA v132 as the reference database [59]. Rarefaction curves were made using the “rarecurve” function of the Vegan package [60]. Four no-template control samples and 3 samples of cecal DNA (M204, M267 and M249) were removed from the analysis because they did not yield more than 1000 sequences and can thus be considered empty. Bacterial richness (Chao1 index) was calculated using the phyloseq package [61] and was compared between different factors (such as age and farm) using a one-way ANOVA and a Tukey honest significant differences post-hoc test from the statistics package. Next, ASVs with an overall summed read count of smaller than 20 were removed. The bacterial diversity was compared between conditions by calculating the Bray Curtis dissimilarities between all samples and constructing non-metric multidimensional scaling plots (NMDS ordination plots) using phyloseq and applying a PERMANOVA (after checking for homoscedasticity) using the “adonis” function of Vegan. Bar plots were made using Phyloseq, and custom adaptations using ggplot2 [62]. Finally, all read counts belonging to ASVs from the same genus were summed, and genera were statistically compared between conditions using a DESeq2 differential expression analysis based on the negative binomial distribution [63]. All genera with an adjusted *p*-value <0.05 (Benjamini-Hochberg correction) were considered significant. The models included Age and Farm as co-variables to correct for confounding. This section may be divided by subheadings. It should provide a concise and precise description of the experimental results, their interpretation, as well as the experimental conclusions that can be drawn.

## Figures and Tables

**Table 1:** Genera were Significantly Different (*p* < 0.05) at Four Weeks of Age versus Three Weeks of Age.

Family	Genus	Occurrence (%) ^a^	Log2-Fold Change ^b^	Adjusted *p*-Value
*Staphylococcaceae*	*Staphylococcus*	1.76	−2.15	2.64 × 10^−6^
*Corynebacteriaceae*	*Corynebacterium*	2.09	−2.06	1.10 × 10^−5^
*Dermabacteraceae*	*Brachybacterium*	0.90	−1.86	0.000304
*Leuconostocaceae*	*Weisella*	0.81	−1.79	0.000212
*Bacteroidaceae*	*Bacteroides*	0.74	−1.00	0.000194
*Lactobacillaceae*	*Lactobacillus*	7.47	−0.43	0.033147
*Ruminococcaceae*	*Subdoligranulum*	1.08	0.25	0.041226
*Lachnospiraceae*	*Fusicatenibacter*	1.21	0.69	9.18 × 10^−8^
*Streptococcaceae*	*Streptococcus*	1.53	2.58	5.49 × 10^−9^

^a^ Mean relative percentage of occurrence across all samples. ^b^ The fold change of age group 4 versus age group 3, expressed as log2value. A negative value means a lower occurrence in age group 4 versus 3, while a positive value means higher occurrence in age group 4 versus 3. Only genera with a mean occurrence >0.5% and an adjusted *p*-value < 0.05 are shown.

**Table 2:** Genera that significantly differed in *Campylobacter*-negative versus *Campylobacter*-positive flocks (*p* < 0.05).

Family	Genus	Occurrence (%) ^a^	Log2-Fold Change ^b^	Adjusted *p*-Value
*Aerococcaceae*	*Globicatella*	0.004	−4.28	2.00 × 10^−6^
*Staphylococcaceae*	*Aliicoccus*	0.005	−3.38	4.55 × 10^−5^
*Family XI_2*	*Gallicola*	0.005	−3.37	0.001126
*Family XI_2*	*Anaerococcus*	0.010	−3.15	3.23 × 10^−5^
*Carnobacteriaceae*	*Atopostipes*	0.005	−3.12	0.000213
*Aerococcaceae*	*Facklamia*	0.122	−2.45	3.23 × 10^−5^
*Dietziaceae*	*Dietzia*	0.038	−2.37	9.76 × 10^−7^
*Streptococcaceae*	*Streptococcus*	1.535	−1.44	0.000291
*Aerococcaceae*	*Aerococcus*	0.426	−1.18	0.025612
*Ruminococcaceae*	*Ruminococcus 1*	0.014	−0.59	0.012061
*Lachnospiraceae*	*Marvinbryantia*	0.125	−0.50	0.00272
*Lachnospiraceae*	*CHKCI001*	0.166	−0.39	0.013882
*Ruminococcaceae*	*Subdoligranulum*	1.084	0.24	0.011057
*Lachnospiraceae*	*Fusicatenibacter*	1.211	0.29	0.011057
*Ruminococcaceae*	*Ruminococcaceae UCG-013*	0.040	0.33	0.004396
*Erysipelotrichaceae*	*Erysipelatoclostridium*	0.187	0.42	7.94 × 10^−5^
*Lachnospiraceae*	*Tyzzerella 3*	0.004	0.47	0.020315
*X Defluviitaleaceae*	*Defluviitaleaceae UCG-011*	0.018	0.48	0.020809
*Eggerthellaceae*	*Gordonibacter*	0.005	0.49	0.016537
*Anaeroplasmataceae*	*Anaeroplasma*	0.029	0.75	0.040975
*Rikenellaceae*	*Alistipes*	0.301	0.88	0.000422
*Enterococcaceae*	*Enterocuccus*	0.280	0.89	0.011057
*Lachnospiraceae*	*Ruminococcus gauveauii group*	0.001	1.07	0.017146
*Lachnospiraceae*	*Lachnoclostridium*	0.006	1.37	0.028353
*Bacillaceae*	*Oceanobacillus*	0.004	1.77	0.022935
*Veillonellaceae*	*Megamonas*	0.725	3.92	9.76 × 10^−7^
*Burkholderiaceae*	*Sutterella*	0.025	5.01	0.002039
*Helicobacteraceae*	*Helicobacter*	0.087	5.07	9.76 × 10^−7^
*Barnesiellaceae*	*Barnesiella*	0.037	5.20	0.000557

^a^ Mean relative percentage of occurrence across all samples. ^b^ The fold change of *Campylobacter*-negative flocks versus *Campylobacter*-positive flocks expressed as log2 values. A negative value means lower occurrence negative versus positive flocks, while a positive value means higher occurrence in negative versus positive flocks. Only significant genera are shown (adjusted *p*-value <0.05).

**Table 3:** Sampling during Vacant Period.

Place of Sampling	Sample	Material Used ^1^	No. Samples ^2^	No. Pools
Anteroom	Boots	Sponge stick	All	1
	Buckets	Sponge stick	All	1
	Door handle	Cotton swab	2	2
	Sink	Sponge stick	1	1
	Flies	Sticky paper	One strip	1
	Boot dip	Sponge stick	1	1
	Floor hygiene barrier	Sponge stick	2	2
Poultry house	Beetles	Sterile tweezers	Undefined	1
	Drinking nipples	Cotton swab	20	4
	Drinking bowl	Cotton swab	20	4
	Cracks in floor and walls	Cotton swab	5	1
	Puddles on floor	Sponge stick	2	2
	Air	Air sampler	4	4
External environment	Puddles on concrete	Sponge stick	2	2
	Cattle and/or swine	Overshoes	One pair	1
	Carcass container	Sponge stick	1	1
	Bird droppings	Sponge stick	1	1

^1^ Material used to sample one broiler flock. ^2^ Number of samples used per sampling place. “All” means that all objects present were sampled (e.g., if two buckets were present, both were sampled).

**Table 4:** Weekly sampling between 3–6 weeks of age.

Place of Sampling	Sample	Material Used ^1^	No. Samples ^2^	No. Pools
Anteroom	Boots	Sponge stick	All	1
	Buckets	Sponge stick	All	1
	Door handle	Cotton swab	2	2
	Sink	Sponge stick	1	1
	Boot dip	Sponge stick	1	1
	Floor hygiene barrier	Sponge stick	2	2
	Wheelbarrow (wheels)	Sponge stick	1	1
Poultry house	Broilers	Cecal droppings	50	5
	Drinking nipples	Cotton swab	20	4
	Drinking bowl	Cotton swab	20	4
	Air	Airsampler	4	4
External environment	Puddles on concrete	Sponge stick	2	2
	Cattle and/or swine	Overshoes	One pair	2
	Carcass container	Sponge stick	1	1
	Bird droppings	Sponge stick	1	1

^1^ Material used to sample one broiler flock. ^2^ Number of samples used per sampling place. “All” means that all objects present were sampled (e.g., if two buckets were present, both were sampled).

## Supplementary Materials

The following are available online at https://www.mdpi.com/2076-0817/10/1/66/s1. Table S1: Occurrence of *Campylobacter* spp. on ten broiler farms for two production cycles (I and II), Figure S1.

## References

[1] EFSA, ECDC (2019). The European Union One Health 2018 Zoonoses Report. EFSA J..

[2] EFSA, ECDC (2011). The European Union Summary Report on Trends and Sources of Zoonoses, Zoonotic Agents and Food-borne Outbreaks in 2009. EFSA J..

[3] Mughini-Gras L., Pijnacker R., Coipan C., Mulder A.C., Veludo A.F., De Rijk S., Van Hoek A.H., Buij R., Muskens G., Koene M. (2020). Sources and transmission routes of campylobacteriosis: A combined analysis of genome and exposure data. J. Infect..

[4] Messens W., Hartnett E., Gellynck X., Viaene J., Halet D., Herman L., Grijspeerdt K. Quantitative Risk Assessment of Human Campylobacteriosis through the Consumption of Chicken Meat in Belgium. Proceedings of the 18th European Symposium on the Quality of Poultry Meat.

[5] Hermans D., Pasmans F., Messens W., Martel A., Van Immerseel F., Rasschaert G., Heyndrickx M., Van Deun K., Haesebrouck F. (2012). Poultry as a Host for the Zoonotic PathogenCampylobacter jejuni. Vector Borne Zoonotic Dis..

[6] Wysok B., Wojtacka J. (2018). Detection of virulence genes determining the ability to adhere and invade in Campylobacter spp. from cattle and swine in Poland. Microb. Pathog..

[7] Plishka M., Sargeant J.M., Greer A.L., Hookey S., Winder C. (2020). The Prevalence of Campylobacter in Live Cattle, Turkey, Chicken, and Swine in the United States and Canada: A Systematic Review and Meta-Analysis. Foodborne Pathog. Dis..

[8] Zweifel C., Scheu K.D., Keel M., Renggli F., Stephan R. (2008). Occurrence and genotypes of Campylobacter in broiler flocks, other farm animals, and the environment during several rearing periods on selected poultry farms. Int. J. Food Microbiol..

[9] Robyn J., Rasschaert G., Pasmans F., Heyndrickx M. (2015). Thermotolerant Campylobacter during Broiler Rearing: Risk Factors and Intervention. Compr. Rev. Food Sci. F.

[10] Agunos A., Waddell L., Léger D., Taboada E. (2014). A Systematic Review Characterizing On-Farm Sources of Campylobacter spp. for Broiler Chickens. PLoS ONE.

[11] Georgiev M., Beauvais W., Guitian J. (2016). Effect of enhanced biosecurity and selected on-farm factors on Campylobacter colonization of chicken broilers. Epidemiol. Infect..

[12] Hald B., Sommer H.M., Skovgård H. (2007). Use of Fly Screens to ReduceCampylobacterspp. Introduction in Broiler Houses. Emerg. Infect. Dis..

[13] Bahrndorff S., Rangstrup-Christensen L., Nordentoft S., Hald B. (2013). Foodborne Disease Prevention and Broiler Chickens with ReducedCampylobacterInfection. Emerg. Infect. Dis..

[14] Han Z., Willer T., Colin P., Pielsticker C., Rychlik I., Velge P., Kaspers B., Rautenschlein S. (2017). Influence of the Gut Microbiota Composition on Campylobacter jejuni Colonization in Chickens. Infect. Immun..

[15] Seliwiorstow T., Duarte A., Baré J., Botteldoorn N., Dierick K., Uyttendaele M., De Zutter L. (2015). Comparison of Sample Types and Analytical Methods for the Detection of Highly Campylobacter-Colonized Broiler Flocks at Different Stages in the Poultry Meat Production Chain. Foodborne Pathog. Dis..

[16] Rasschaert G., Houf K., Van Hende J., De Zutter L. (2007). Investigation of the concurrent colonization with Campylobacter and Salmonella in poultry flocks and assessment of the sampling site for status determination at slaughter. Vet. Microbiol..

[17] Herman L., Heyndrickx M., Grijspeerdt K., Vandekerchove D., Rollier I., De Zutter L. (2003). Routes for Campylobacter contamination of poultry meat: Epidemiological study from hatchery to slaughterhouse. Epidemiol. Infect..

[18] Fernandez H., Vergara M., Tapia F. (1985). Dessication resistance in thermotolerant campylobacter species. Infection.

[19] Van Gerwe T., Miflin J.K., Templeton J.M., Bouma A., Wagenaar J.A., Jacobs-Reitsma W.F., Stegeman A., Klinkenberg D. (2008). Quantifying Transmission of *Campylobacter jejuni* in Commercial Broiler Flocks. Appl. Environ. Microbiol..

[20] Stern N.J., Cox N.A., Musgrove M.T., Park C.M. (2001). Incidence and Levels of Campylobacter in Broilers After Exposure to an Inoculated Seeder Bird. J. Appl. Poult. Res..

[21] Messens W., Herman L., De Zutter L., Heyndrickx M. (2009). Multiple typing for the epidemiological study of contamination of broilers with thermotolerant Campylobacter. Vet. Microbiol..

[22] Sparks N. (2009). The role of the water supply system in the infection and control of Campylobacter in chicken. World Poult. Sci. J..

[23] Newell D.G., Fearnley C. (2003). Sources of Campylobacter Colonization in Broiler Chickens. Appl. Environ. Microbiol..

[24] Gregory E., Barnhart H., Dreesen D.W., Stern N.J., Corn J.L. (1998). Epidemiological study of Campylobacter spp. in broilers: Source, time of colonization, and prevalence. Avian Dis..

[25] Evans S.J. (1992). Introduction and spread of thermophilic campylobacters in broiler flocks. Vet. Rec..

[26] Søndergaard M.S.R., Josefsen M.H., Löfström C., Christensen L.S., Wieczorek K., Osek J., Hoorfar J. (2014). Low-Cost Monitoring of Campylobacter in Poultry Houses by Air Sampling and Quantitative PCR. J. Food Prot..

[27] Carrique-Mas J., Bryant J.E., Cuong N.V., Hoang N.V.M., Campbell J., Dung T.T.N., Duy D., Hoa N.T., Thompson C., Hien V.V. (2013). An epidemiological investigation ofCampylobacterin pig and poultry farms in the Mekong delta of Vietnam. Epidemiol. Infect..

[28] Ragimbeau C., Schneider F., Losch S., Even J., Mossong J. (2008). Multilocus Sequence Typing, Pulsed-Field Gel Electrophoresis, and *fla* Short Variable Region Typing of Clonal Complexes of *Campylobacter jejuni* Strains of Human, Bovine, and Poultry Origins in Luxembourg. Appl. Environ. Microbiol..

[29] Patriarchi A., Fox Á., Maunsell B., Fanning S., Bolton D. (2011). Molecular Characterization and Environmental Mapping of Campylobacter Isolates in a Subset of Intensive Poultry Flocks in Ireland. Foodborne Pathog. Dis..

[30] Rapp D., Ross C.M., Pleydell E.J., Muirhead R.W. (2012). Differences in the Fecal Concentrations and Genetic Diversities of *Campylobacter jejuni* Populations among Individual Cows in Two Dairy Herds. Appl. Environ. Microbiol..

[31] Hertogs K., Heyndrickx M., Gelaude P., De Zutter L., Dewolf J., Rasschaert G. (2020). The effect of partial depopulation on Campylobacter introduction in broiler houses. Poult. Sci..

[32] Rasschaert G., De Zutter L., Herman L., Heyndrickx M. (2020). Campylobacter contamination of broilers: The role of transport and slaughterhouse. Int. J. Food Microbiol..

[33] Ridley A., Morris V., Gittins J., Cawthraw S., Harris J., Edge S., Allen V. (2011). Potential sources of Campylobacter infection on chicken farms: Contamination and control of broiler-harvesting equipment, vehicles and personnel. J. Appl. Microbiol..

[34] Chaisowwong W., Kusumoto A., Hashimoto M., Harada T., Maklon K., Kawamoto K. (2012). Physiological Characterization of *Campylobacter jejuni* under Cold Stresses Conditions: Its Potential for Public Threat. J. Vet. Med. Sci..

[35] Gong J., Yu H., Liu T., Gill J., Chambers J., Wheatcroft R., Sabour P. (2008). Effects of zinc bacitracin, bird age and access to range on bacterial microbiota in the ileum and caeca of broiler chickens. J. Appl. Microbiol..

[36] Barnes E.M., Impey C.S., Stevens B. (1979). Factors affecting the incidence and anti-salmonella activity of the anaerobic caecal flora of the young chick. J. Hyg..

[37] Sergeant M.J., Constantinidou C., Cogan T.A., Bedford M.R., Penn C.W., Pallen M.J. (2014). Extensive Microbial and Functional Diversity within the Chicken Cecal Microbiome. PLoS ONE.

[38] Walugembe M., Hsieh J.C.F., Koszewski N.J., Lamont S.J., Persia M.E., Rothschild M.F. (2015). Effects of dietary fiber on cecal short-chain fatty acid and cecal microbiota of broiler and laying-hen chicks. Poult. Sci..

[39] Polansky O., Sekelova Z., Faldynova M., Sebkova A., Sisak F., Rychlik I. (2016). Important Metabolic Pathways and Biological Processes Expressed by Chicken Cecal Microbiota. Appl. Environ. Microbiol..

[40] González-Fandos E., Maya N., Pérez-Arnedo I. (2015). Effect of propionic acid on *Campylobacter jejuni* attached to chicken skin during refrigerated storage. Int. Microbiol..

[41] Scupham A.J., Jones J.A., Rettedal E.A., Weber T.E. (2010). Antibiotic Manipulation of Intestinal Microbiota to Identify Microbes Associated with *Campylobacter jejuni* Exclusion in Poultry. Appl. Environ. Microbiol..

[42] Guerin M.T., Martin W., Reiersen J., Berke O., McEwen S.A., Bisaillon J.-R., Lowman R. (2007). A farm-level study of risk factors associated with the colonization of broiler flocks with *Campylobacter* spp. in Iceland, 2001–2004. Acta Vet. Scand..

[43] Hald B., Skovgård H., Pedersen K., Bunkenborg H. (2008). Influxed Insects as Vectors for *Campylobacter jejuni* and Campylobacter coli in Danish Broiler Houses. Poult. Sci..

[44] Nather G., Alter T., Martin A., Ellerbroek L. (2009). Analysis of risk factors for Campylobacter species infection in broiler flocks. Poult. Sci..

[45] Battersby T., Whyte P., Bolton D. (2016). The pattern of Campylobacter contamination on broiler farms; external and internal sources. J. Appl. Microbiol..

[46] Høg B.B., Sommer H., Larsen L., Sørensen A., David B., Hofshagen M., Rosenquist H. (2016). Farm specific risk factors for Campylobacter colonisation in Danish and Norwegian broilers. Prev. Vet. Med..

[47] Sommer H., Høg B.B., Larsen L., Sørensen A.I.V., Williams N.J., Merga J., Cerdà-Cuéllar M., Urdaneta S., Dolz R., Wieczorek K. (2016). Analysis of farm specific risk factors for Campylobacter colonization of broilers in six European countries. Microb. Risk Anal..

[48] KMI. https://www.meteo.be/resources/climateReportWeb/klimatologisch_jaaroverzicht_2018.pdf.

[49] Linton D., Lawson A.J., Owen R.J., Stanley J. (1997). PCR detection, identification to species level, and fingerprinting of *Campylobacter jejuni* and *Campylobacter coli* direct from diarrheic samples. J. Clin. Microbiol..

[50] Linton D., Owen R., Stanley J. (1996). Rapid identification by PCR of the genus Campylobacter and of five Campylobacter species enteropathogenic for man and animals. Res. Microbiol..

[51] Nachamkin I., Bohachick K., Patton C.M. (1993). Flagellin gene typing of *Campylobacter jejuni* by restriction fragment length polymorphism analysis. J. Clin. Microbiol..

[52] PulseNet (2017). Standard Operating Procedure for PulseNet PFGE of *Campylobacter jejuni*. https://www.cdc.gov/pulsenet/pdf/campylobacter-pfge-protocol-508c.pdf.

[53] Klindworth A., Pruesse E., Schweer T., Peplies J., Quast C., Horn M., Glöckner F.O. (2012). Evaluation of general 16S ribosomal RNA gene PCR primers for classical and next-generation sequencing-based diversity studies. Nucleic Acids Res..

[54] R Core Team (2019). R: A Language and Environment for Statistical Computing.

[55] RStudio Team (2019). RStudio: Integrated Development for R.

[56] Morgan M., Anders S., Lawrence M., Aboyoun P., Pagès H., Gentleman R. (2009). ShortRead: A bioconductor package for input, quality assessment and exploration of high-throughput sequence data. Bioinformatics.

[57] Callahan B.J., McMurdie P.J., Rosen M.J., Han A.W., Johnson A.J., Holmes S.P. (2016). DADA2: High resolution sample inference from amplicon data. Nat. Methods.

[58] Murali A., Bhargava A., Wright E.S. (2018). IDTAXA: A novel approach for accurate taxonomic classification of microbiome sequences. Microbiome.

[59] Yilmaz P., Parfrey L.W., Yarza P., Gerken J., Pruesse E., Quast C., Schweer T., Peplies J., Ludwig W., Glöckner F.O. (2014). The SILVA and “All-species Living Tree Project (LTP)” taxonomic frameworks. Nucleic Acids Res..

[60] Oksanen J., Blanchet F.G., Friendly M., Kindt R., Legendre P., McGlinn D., Minchin P.R., O’Hara R.B., Simpson G.L., Solymos P. (2019). Vegan: Community Ecology Package.

[61] McMurdie P.J., Holmes S. (2013). phyloseq: An R Package for Reproducible Interactive Analysis and Graphics of Microbiome Census Data. PLoS ONE.

[62] Wickham H. (2016). ggplot2 Elegant Graphics for Data Analysis. J. R. Stat. Soc. Ser. A.

[63] Love M.I., Huber W., Anders S. (2014). Moderated estimation of fold change and dispersion for RNA-seq data with DESeq2. Genome Biol..

